# Safety and long-term improvement of mesenchymal stromal cell infusion in critically COVID-19 patients: a randomized clinical trial

**DOI:** 10.1186/s13287-022-02796-1

**Published:** 2022-03-21

**Authors:** Carmen Lúcia Kuniyoshi Rebelatto, Alexandra Cristina Senegaglia, Claudio Luciano Franck, Debora Regina Daga, Patrícia Shigunov, Marco Augusto Stimamiglio, Daniela Boscaro Marsaro, Bruna Schaidt, Andressa Micosky, Ana Paula de Azambuja, Cleverson Alex Leitão, Ricardo Rasmussen Petterle, Valderez Ravaglio Jamur, Isadora May Vaz, Antônio Paulo Mallmann, Hipólito Carraro Junior, Eduardo Ditzel, Paulo Roberto Slud Brofman, Alejandro Correa

**Affiliations:** 1 Core for Cell Technology, School of Medicine, Pontifícia Universidade Católica Do Paraná, 1155 Imaculada Conceição Street, Prado Velho, Curitiba, PR 80215-901 Brazil; 2 Complexo Hospital de Clínicas, Universidade Federal do Paraná, Curitiba, PR Brazil; 3 National Institute of Science and Technology for Regenerative Medicine, INCT-REGENERA, Rio de Janeiro, Brazil; 4 Laboratory of Basic Biology of Stem Cells, Carlos Chagas Institute, Fiocruz-Paraná, Curitiba, PR Brazil; 5 Statistics, Health Sciences Sector, Federal University of Paraná, Curitiba, PR Brazil; 6 Hospital e Maternidade Brígida, Curitiba, PR Brazil; 7 Hospital Nossa Senhora do Pilar, Curitiba, PR Brazil

**Keywords:** COVID-19, Mesenchymal stromal cells, Postacute sequelae, Cell therapy

## Abstract

**Background:**

COVID-19 is a multisystem disease that presents acute and persistent symptoms, the postacute sequelae (PASC). Long-term symptoms may be due to consequences from organ or tissue injury caused by SARS-CoV-2, associated clotting or inflammatory processes during acute COVID-19. Various strategies are being chosen by clinicians to prevent severe cases of COVID-19; however, a single treatment would not be efficient in treating such a complex disease. Mesenchymal stromal cells (MSCs) are known for their immunomodulatory properties and regeneration ability; therefore, they are a promising tool for treating disorders involving immune dysregulation and extensive tissue damage, as is the case with COVID-19. This study aimed to assess the safety and explore the long-term efficacy of three intravenous doses of UC-MSCs (umbilical cord MSCs) as an adjunctive therapy in the recovery and postacute sequelae reduction caused by COVID-19. To our knowledge, this is one of the few reports that presents the longest follow-up after MSC treatment in COVID-19 patients.

**Methods:**

This was a phase I/II, prospective, single-center, randomized, double-blind, placebo-controlled clinical trial. Seventeen patients diagnosed with COVID-19 who require intensive care surveillance and invasive mechanical ventilation—critically ill patients—were included. The patient infusion was three doses of 5 × 10^5^ cells/kg UC-MSCs, with a dosing interval of 48 h (*n* = 11) or placebo (*n* = 6). The evaluations consisted of a clinical assessment, viral load, laboratory testing, including blood count, serologic, biochemical, cell subpopulation, cytokines and CT scan.

**Results:**

The results revealed that in the UC-MSC group, there was a reduction in the levels of ferritin, IL-6 and MCP1-CCL2 on the fourteen day. In the second month, a decrease in the levels of reactive C-protein, D-dimer and neutrophils and an increase in the numbers of TCD3, TCD4 and NK lymphocytes were observed. A decrease in extension of lung damage was observed at the fourth month. The improvement in all these parameters was maintained until the end of patient follow-up.

**Conclusions:**

UC-MSCs infusion is safe and can play an important role as an adjunctive therapy, both in the early stages, preventing severe complications and in the chronic phase with postacute sequelae reduction in critically ill COVID-19 patients.

*Trial registration* Brazilian Registry of Clinical Trials (ReBEC), UTN code-U1111-1254-9819. Registered 31 October 2020—Retrospectively registered, https://ensaiosclinicos.gov.br/rg/RBR-3fz9yr

**Supplementary Information:**

The online version contains supplementary material available at 10.1186/s13287-022-02796-1.

## Introduction

COVID-19 has rapidly spread [1–4] and was officially declared a pandemic by the World Health Organization (WHO) in March 2020 [4, 5]. By July 2020, approximately 1.4 million cases were reported worldwide, while over 1 million were reported only in the Americas [4]. Today, the world has reported more than 402 million cases with over 5 million deaths [4]. Brazil has been particularly affected by the COVID-19 pandemic. A total of over 26 million cases (almost 8% of the total cases in the world) have already been reported, with daily new cases reaching 298,000 [4]. The highest daily death report was on April 8th, 2021, with 4195 deaths, and Brazil has arduously accumulated over 633,000 deaths until February 2022 [4].

Mortality due to COVID-19 varies according to many factors, such as case definition [6], population immunization rate, access to health care, age, severity of disease and comorbidities [7]. From July to August 2020, period in which this study was carried out, the mortality in the Americas varied between 2.66 and 3.09% among all reported cases [4], while in Paraná (Brazil), where the investigation has taken place, mortality was reported to be 2.5% [8, 9]. A systematic review and meta-analysis suggested that mortality among patients in an intensive care unit (ICU) decreased over time from above 50% in March to approximately 40% at the end of May 2020 [10], suggesting that improvement of COVID-19 knowledge and better intensive care have a direct impact on survival. Among patients admitted to the ICU, both noninvasive and invasive mechanical ventilation (IMV) was associated with higher mortalities, with statistically significant hazard ratios of 2.36 and 3.77, respectively [7]. In Brazil, COVID-19 mortality among patients admitted was 24.4%, and approximately 55.7% of the hospitalized patients needed intensive care [11]. Patients admitted to the ICU and on MV in Brazil were shown to have higher mortality rates, as 28-day mortality has been reported to range from 56.3 to 61.5% [12]. Age, cardiovascular disease, neurological disorders and pneumopathies were related to a higher probability of death [13].

COVID-19 is a multisystem disease, and the features at the beginning are fever, cough and headache. The second phase exhibits high-grade fever, difficulty breathing and pneumonia-like symptoms [14]. The progression to the third stage is mediated by inflammatory cytokines and chemokines and massive infiltration of inflammatory cells, causing cytokine release syndrome (CRS). This syndrome induces pulmonary edema, severe acute respiratory distress syndrome (SARS), vascular dysfunction through microvascular thrombosis [15], tissue remodeling and fibrosis [16] and multiple-organ dysfunction syndrome (MODS), which ultimately lead to death [17, 18].

Currently, one of the major concerns is the persistent symptoms after apparent resolution from COVID-19. Patients who develop chronic symptoms after acute COVID-19 are diagnosed with long COVID or postacute sequelae of COVID-19 (PASC) [19]. Long-term symptoms may be due to organ or tissue injury caused by SARS-CoV-2 or associated clotting or inflammatory processes during acute COVID-19 [20]. PASC prevalence is high and represents very significant public health and economic consequences [21–24]. The dynamic equilibrium maintained by innate and adaptive immunity is essential to avoid the progression of COVID-19 [25]. The formation of an appropriate innate immune response in the early stages of the disease, followed by an effective adaptive immune response, limits the virus spreading and prevents tissue damage. In patients infected with SARS-CoV-2, the plasma levels of inflammatory cytokines increase [26]. In contrast, there is a significant decrease in the total number of T cells, T helper (CD4) and cytotoxic suppressor (CD8) T cells, NK cells and regulatory T cells, compromising the immune system [27]. Reduced expression of memory T cells may be a plausible explanation for the increased reinfection rates by SARS-CoV-2 [28].

Various treatment strategies are being chosen to combat this disease; however, no definite therapy has been proven to completely control the cytokine storm and to restore the organ damage caused by COVID-19 infection. PASC patients who develop chronic symptoms after hospitalization for acute COVID-19 may be more likely to suffer from injury to one or more body sites [29]. Hence, different therapeutic approaches for COVID-19 should not only eliminate the virus and treat CRS but also accelerate recovery and minimize chronic symptoms.

Mesenchymal stromal cells (MSCs) are known for their immunomodulatory properties that occur directly via interaction with host immune cells or indirectly through paracrine secretion or endocrine factors, which act on nearby cells or travel through the blood to exert their effects [30, 31]. Additionally, these cells can produce an antimicrobial effect [32–34], antiapoptotic effect and regeneration. MSCs can be more beneficial than other anti-inflammatory agents because they can provide immunomodulatory effects based on host cells [35]. In addition, MSCs can reduce inflammation, prevent fibrosis of tissues, enable reversal of lung dysfunction, protect and repair alveolar cells and aid in the regeneration of damaged tissue, which can be significantly beneficial for COVID-19-associated organ [36, 37]. MSCs do not express angiotensin-*converting* enzyme 2 (ACE2) and transmembrane serine protease 2 (TMPRSS2), which specifically recognize and bind with the spike (S) protein of SARS-CoV-2. Since the S protein plays an essential role in virus infection and transmission [38, 39], MSCs cannot be infected with SARS-CoV-2 [40]. Umbilical cord MSCs (UC-MSCs) can be obtained using a noninvasive method; they are easily isolated and have great potential for cell expansion, and cells from young donors are less susceptible to oxidative damage [41, 42]. Intravenously infused UC-MSCs enable metabolism's first-pass effect, where MSCs are entrapped in the lung vasculature [43, 44]. Therefore, they may be effective in treating lung diseases.

MSCs are a promising tool for treating disorders involving immune dysregulation and extensive tissue damage, as is the case with COVID-19. Studies have shown that intravenous MSC infusion in patients with COVID-19 is safe and well tolerated. It prevents or reduces ARDS and other serious complications, decreases inflammatory cytokines and mortality and in some cases improves pulmonary functions [40, 45–58]. MSCs, due to their immunomodulatory, anti-inflammatory and tissue regeneration potential, could be a new therapeutic strategy to treat critically ill COVID-19 patients and minimize sequelae symptoms.

The present study aimed to assess the safety and explore the long-term efficacy of UC-MSCs infusion as an adjunctive therapy in the recovery and PASC reduction caused by COVID-19.

## Material and methods

### Study design and patient population

**Fig. 1:**
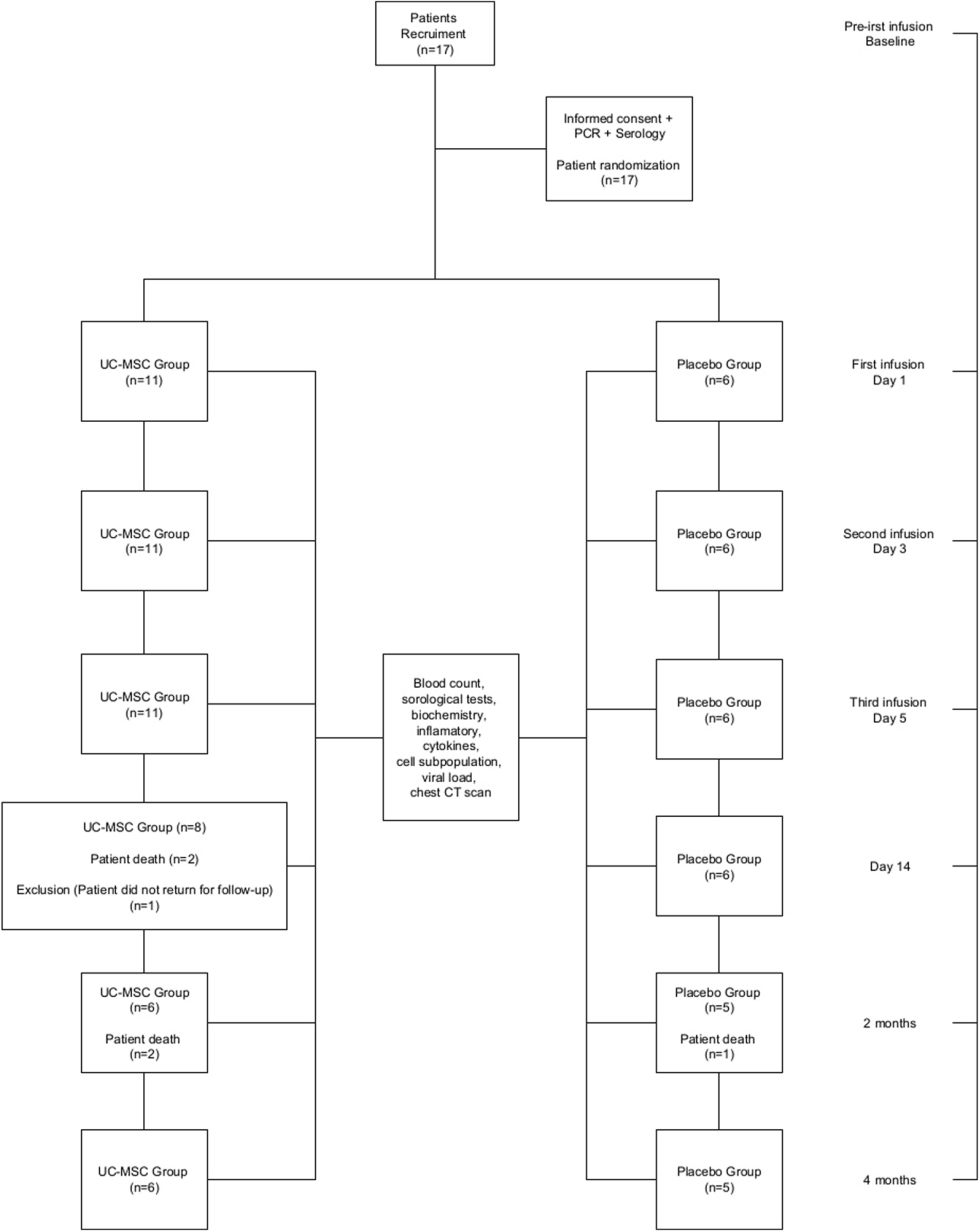
Flow chart for patient enrollment, intervention and follow-up

This study was a phase I/II, prospective, single-center, randomized, double-blind, placebo-controlled clinical trial. The subjects were recruited from the Complexo Hospital de Clínicas, Universidade Federal do Paraná, a referral public hospital for the treatment of patients with COVID-19, and UC-MSCs were processed at Core for Cell Technology (CCT) from the Pontificia Universidade Católica do Paraná (PUCPR). UCs were obtained from full-term neonates, and the mother of the donor signed an informed consent form approved by the institutional review board. Written informed consent, for the collection and publication of clinical data, was also obtained from the patients’ parents due to the impossibility of the patients themselves, who were sedated and MV, to sign the authorization to participate in research (Fig. 1). The experimental design was conducted in accordance with the Helsinki Declaration for human studies and was approved by the Ethics Committee (CAAE: 30833820.8.0000.0020). This study was registered in the Brazilian Registry of Clinical Trials (ReBEC), UTN code-U1111-1254-9819. Registered 31 October 2020—Retrospectively registered, https://ensaiosclinicos.gov.br/rg/RBR-3fz9yr.

Patients over 18 years old, diagnosed with COVID-19 (as evaluated by reverse-transcription polymerase chain reaction (RT-PCR) test confirming infection with SARS-CoV-2), SARS associated-coronavirus*,* who require intensive care surveillance and IMV—critically ill patients (WHO ordinal scale score 6 and 7), arterial oxygen partial pressure (PaO_2_)/oxygen absorption concentration (FiO_2_) ≤ 300 mmHg, were eligible for inclusion. Exclusion criteria were: use of any investigational products, previous or current history of malignancy under treatment; preexisting thromboembolic disease; concomitant infection of human immunodeficiency virus (HIV) or tuberculosis infection; pregnancy; preexisting transplant or use of immunosuppressive therapy; inability to provide informed consent and greater than 72 h of ICU admission.

The primary outcome of this study was the safety of allogenic UC-MSC infusion after the observation of infusional reactions and adverse events (AEs). The second outcome included patient recovery demonstrated through viral load, blood tests and plasma levels of inflammatory cytokines, peripheral blood mononuclear cell (PBMC) assessment of T cell populations and PASC reduction evaluated by biochemical markers and CT scan.

The primary safety endpoints encompassed the occurrence of prespecified infusion-associated AEs within 24 h after intravenous administration of UC-MSCs or placebo. According to the Common Terminology Criteria for Adverse Events version 5.0, it was assessed by recording all AEs based on duration, intensity and possible association with the treatment under study. Investigators conducted assessments for the presence of any AEs from enrollment throughout the study. The secondary endpoint was exploratory efficacy defined by clinical outcomes, changes in viral load, inflammatory, immunological and biochemical biomarkers and acute lung injury (ALI) score.

Seventeen eligible patients were enrolled in an approximate 1:2 randomization, according to the randomization table generated by the R program version 4.1.2 (R Core Team, 2021). The treatment were IV infusion of three doses of 5 × 10^5^ cells/kg UC-MSCs, a dosing interval of 48 h (*n* = 11), or placebo (*n* = 6). Concomitant corticosteroids and anticoagulants were allowed, and conventional treatment was performed together with the infusion of cells during the study period. All patients were assessed at baseline and the pre-established follow-up points on days 2, 4, 6 and 14, as well as at 2 and 4 months post-infusion. These evaluations consisted of a clinical assessment, viral load, laboratory testing, including blood count, serologic, biochemical, cell subpopulation, cytokine and CT scan evaluations.

### Advanced therapy product

The study was conducted following the Good Clinical Practice Guidelines for Advanced Therapy Products (ATP) (RDC 508/2021). One week before UC collection, healthy donors provided written informed consent, and serology for infectious diseases and RT–PCR for COVID-19 were performed.

UCs were obtained from full-term newborns by cesarean section and they were aseptically stored in sterile Iscove's modified Dulbecco's medium (IMDM) (Gibco BRL, Grand Island, NY) supplemented with 100 U/mL penicillin and 100 µg/mL streptomycin (Gibco BRL, Grand Island, NY). The umbilical cord was washed three times with phosphate buffered saline (PBS) (Gibco BRL, Grand Island, NY) and antibiotics, sectioned into small fragments (1–2 mm^2^ pieces) and centrifuged at 280* g* for 10 min. After removing the supernatant fraction, the precipitate was washed with IMDM and centrifuged at 280* g* for 10 min. The tissue was treated with 0.1% collagenase type II (Sigma, St Louis, MO, USA) at 37 °C for 16 h, washed and further digested with 0.25% trypsin–EDTA (Gibco, Grand Island, NY, USA) at 37 °C for 15 min. Fetal bovine serum (FBS) (HyClone™, South Logan, USA) was added to the MSCs to neutralize the excess trypsin [59].

Cells were plated in 75 cm^2^ culture flasks (Greiner Bio-One, Kremsmünster, AT) with IMDM supplemented with 20% FBS, 100 U/mL penicillin and 100 mg/mL streptomycin and incubated at 37 °C and 5% CO_2_. At 96 h, nonadherent cells were removed and washed with PBS, and the culture medium was replaced with fresh medium every three days. When the cell culture reached 70–80% confluence, cells were detached by treatment with 0.25% trypsin–EDTA and replated at a density of 8000 cells/cm^2^ into 150 cm^2^ culture flasks. At passage 3 (P3), cytogenetic analysis was performed. UC-MSCs were harvested and cryopreserved using a rate-controlled freezer at a final concentration of 10% dimethyl sulfoxide (Origen, Texas, USA) and 90% FBS. Four days before infusion, cells were thawed and replated at a density of 8000 cells/cm^2^ into 150 cm^2^ culture flasks. When the number of cells was sufficient for administration, confluent UC-MSCs were detached with 0.25% trypsin–EDTA and washed twice with saline, and samples were collected for quality control. This control includes viability and cell surface markers by flow cytometry [60], cytogenetics analysis by GTG-banding method [61], microbiological tests (Bact/Alert 3D, Biomerieux, Durham, USA), endotoxin (Endosafe ™ PTS, Charles River, Charleston, USA) and Mycoplasma (KIT MycoAlert ™ PLUS Mycoplasma Detection, Lonza, Rockland, USA), according to the manufacturer’s instructions. These quality control tests were performed before each batch of cells was released. For infusion, 5 × 10^5^ UC-MSCs/kg of body weight were resuspended in a final volume of 30 mL of vehicle solution composed of saline (JP, Ribeirão Preto, Brazil), 5% Anticoagulant Citrate Dextrose (ACD) (JP, Ribeirão Preto, Brazil) and 20% albumin (Blau Farmacêutica, São Paulo, Brazil). The placebo group received a vehicle solution. Cells were infused between P3 and P5.

The release criteria for the clinical use of UC-MSCs included the absence of contamination with pathogenic microorganisms (bacteria, Mycoplasma and fungi) or endotoxin (≤ 0.5 EU/mL), cell viability ≥ 70%, identity and purity pattern characterized by positive (≥ 95%) of CD73, CD90, CD105 and CD29 and negative expression (≤ 2%) of CD45, CD34, CD14, CD19 and HLA-DR.

### RNA extraction and RT–qPCR

RNA was extracted from 140 µl pulmonary aspirate samples from COVID-19 patients by using a QIAamp Viral RNA Kit (Qiagen, Hilden, Germany). RT–qPCR was performed using the GoTaq Probe 1-Step RT–qPCR System (Promega, Madison, EUA) according to the manufacturer’s instructions. A 10 μL reaction contained 2.5 μL of total RNA, 5 μL of GoTaq® Probe qPCR Master Mix with dUTP (2X), 0,2 μl GoScript™ RT Mix for 1-Step RT–qPCR, 600 nM RdRp primer Forward, 800 nM RdRP primer Reverse, 100 nM RdRP probe and 50 nM RNApol probe and primer Forward and Reverse. The primer and probe sequences were as follows: (1) RdRP (NC_045512.2): RdRp_SARSr-F GTGARATGGTCATGTGTGGCGG; RdRp_SARSr-R CARATGTTAAASACACTATTAGCATA and RdRp_SARSr-P2 FAM-CAGGTGGAACCTCATCAGGAGATGC-BBQ [62]; (2) Homo sapiens RNA polymerase II subunit A (POLR2A) (NM_000937.5): RNA Pol-F TGGACAGGCAAGCAAATCTTC; RNA Pol-R AAGGGCCACTGTCTTCATCATC and RNA Pol-Probe Cy5-TACCCACAGCACCCATCCCGATG-BBQ. *R is G/A; S is G/C. FAM: 6-carboxyfluorescein; Cy-5: Cyanine-5; BBQ: blackberry quencher. The reactions were performed in triplicate using the LightCycler system (Roche, Basiléia, Suíça), and thermal cycling was performed at 45 °C for 15 min for reverse transcription, followed by 95 °C for 3 min and then 45 cycles of 95 °C for 15 s and 58 °C for 45 s. For viral quantification analysis, the Cq results for the RdRP gene were normalized based on POLR2A quantity. The relative quantification of RdRP was calculated in relation to the preinfusion time of MSCs or placebo using ΔΔCq methods. The angular coefficient and *R*^2^ were established after linearizing the data (ln (*x*)) and obtaining the linear equation of each patient.

### Multiparametric flow cytometry

Multiparametric flow cytometry (MFC) was performed on all patients at baseline and on specific days after infusion (days 2, 4, 6 and 14, as well as at 2 and 4 months). Absolute leukocyte counts were performed using a Sysmex XN-3000 counter at the time of MFC analysis.

Commercial antibodies were used to analyze the expression of the cell surface markers CD3, CD4, CD8, CD19, CD38, CD127, CD25 and HLA-DR (Becton Dickinson, San Diego, USA). Immunophenotypic characterization of peripheral blood (PB) lymphocytes was performed with conventional staining sample preparation techniques [63]. A total of 1,000,000 cells/events per tube were acquired using a FACSCanto II® flow cytometer (Becton Dickinson, Franklin Lakes, USA) and Infinicyt™ software (Cytognos, Salamanca, Spain—version 2.0) for flow cytometry analyses. The analysis protocol included removal of threshold debris, and lymphocytes were initially identified based on low frontal (FSC) and side scatter (SSC) and strong CD45 staining. The frequency and cell number of total, CD4+ and CD8+ T cells, as well as B (CD19+) and plasmablasts (CD19+ CD38++) in patients, were determined using a Boolean strategy [64]. CD19-positive staining identified B cells, and strong CD38 positivity in B cells was used to identify the plasmablast population (CD19++ CD38++). To identify CD4 regulatory T lymphocytes (Tregs), it was used CD25 positivity and CD127 negativity in the CD4 lymphocyte gate.

### Analysis of inflammatory cytokines, chemokines and growth factors in peripheral blood plasma

Blood samples were collected in EDTA Vacutainer® tubes (BD Biosciences, Curitiba, PR) and immediately transported to the laboratory for processing. Plasma was obtained by centrifugation (1600* g* for 15 min at 4 °C), divided into aliquots and stored at − 80 °C until analysis.

The BD™ Cytometric Bead Array System (CBA Flex Set System, BD Biosciences, San Diego, EUA) was used to determine plasma levels of a set of inflammatory cytokines, chemokines and growth factors, including granulocyte–macrophage colony‐stimulating factor (GM‐CSF), interleukin (IL) -2, IL‐6, IL‐7, IL‐8, tumor necrosis factor (TNF) α, monocyte chemoattractant protein-1 (MCP1/CCL2) and macrophage inflammatory protein 1-alpha (MIP1a/CCL3), according to the manufacturer's recommendations. The assay was performed at preinfusion and on days 2, 4, 6 and 14, as well as 2 and 4 months post-infusion. All samples were measured in duplicate. Standard curves for each cytokine were generated using the premixed lyophilized standards provided in the kits. The cytokine concentrations in samples were determined by measuring their fluorescent intensities and referencing from the appropriate standard curve. Data were analyzed using the FlowJo™ v.10 software.

### Image evaluation

All scans were obtained using a 64-row multidetector scanner (Toshiba Aquilion 64 TSX 101A, Tokyo, Japan). Chest CT evaluation was blinded and the following characteristics were assessed: ground-glass opacities, linear opacities, consolidation, interlobular septal thickening, crazy-paving pattern, subpleural lines, bronchial wall thickening, lymph node enlargement and pleural effusion. Lesions were quantified by assigning a score to all abnormal areas involved [65]. Each lobe was assigned a score of 0 (0% involvement), 1 (1–25% involvement), 2 (26–50% involvement), 3 (51–75% involvement) or 4 (76–100% involvement). The total score was the sum of all lobes, ranging from 0 to 25.

### Statistics

In the descriptive analysis, it was used absolute and relative frequencies for categorical variables, and for quantitative variables, it was calculated the average values with their respective standard deviations. To investigate the change in response variable over time, it was used the framework of Gaussian copula marginal regression models [66] for longitudinal data analysis. First, it was select the probability distribution for the response variable from either Gaussian or gamma. Thereafter, it was select the available correlation structure: (1) independent, (2) exchangeable, (3) autoregressive of order 1 (AR1) and (4) moving average of order 1 (MA1). Next, it was investigated the interaction effect between the group (placebo or UC-MSCs) and the evaluation times (time) that were treated as a factor with seven levels (baseline, 2, 4, 6 and 14 days, 2 and 4 months). When the interaction was not significant (*p* > 0.05), it was used the additive effect on the linear predictor. On the other hand, when the interaction effect was significant, it was conducted a multiple comparison test, where the *p* values were obtained through Bonferroni correction [67]. It was adopted the Akaike (AIC) and Bayesian (BIC) information criteria and the maximized value of the log-likelihood function (logLik) to select the probability distribution for each response variable and the structure for the correlation matrix. The statistical data analysis was performed in R software version 4.1.2 [68] using the R package GCMR [69].

## Results

### Advanced therapy product

In this study, samples from four UC donors were used, and all of them were negative for RT-PCR tests for COVID-19 and serology for infectious diseases. In this clinical trial, fresh infused cells were used, and the interval between product release and patient IV infusion was up to three hours.

**Fig. 2:**
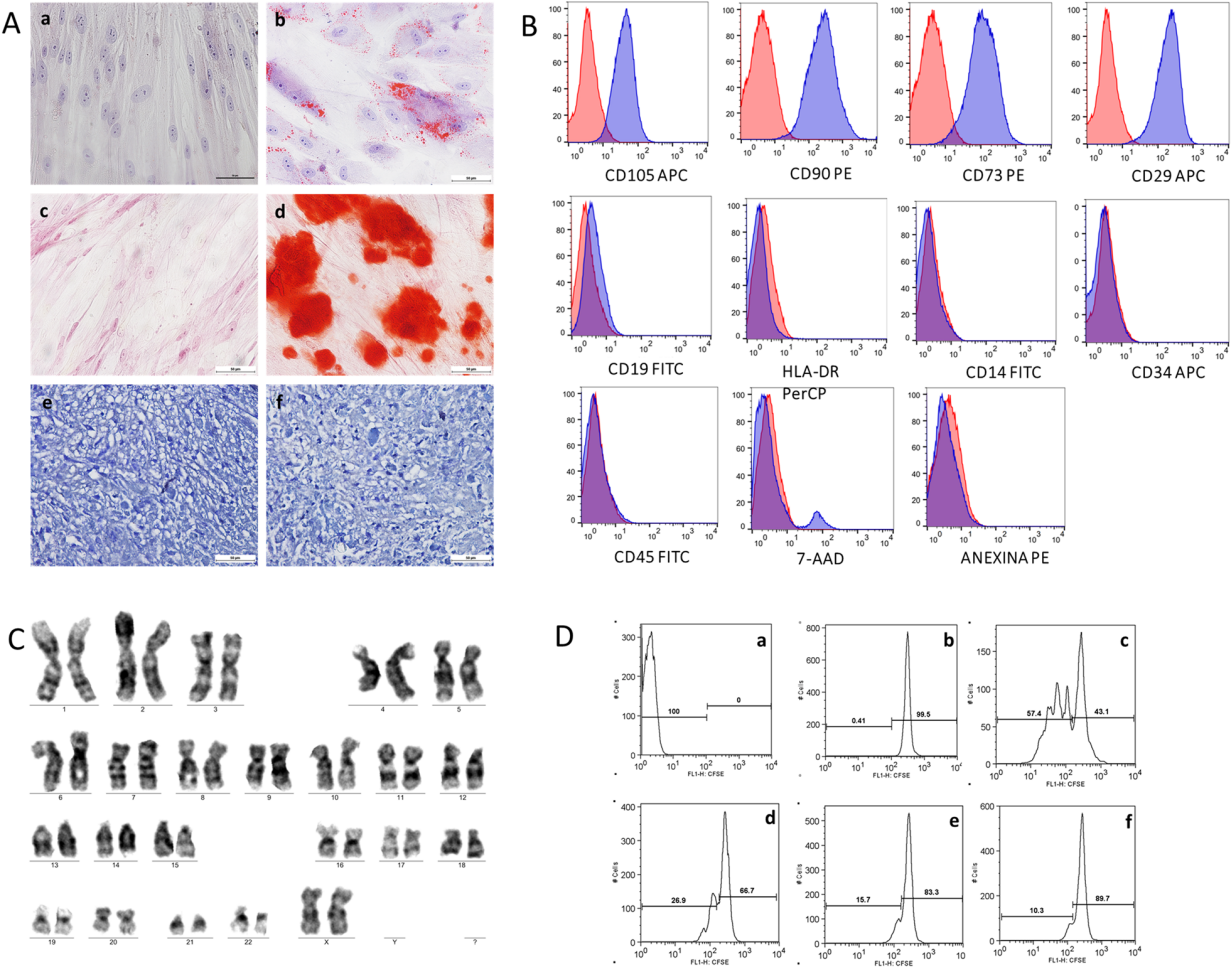
Characterization and quality control for UC-MSC. **A** Representative image of cell differentiation. (a, c, e) Control cells; (b) Cells differentiated into adipocytes characterized by the presence of lipidic vacuoles stained with Oil Red O; (d) Cells differentiated into osteoblasts characterized by the presence of calcium deposits stained with Alizarin Red S (red); (f) Presence of vacuoles around young chondrocytes and proteoglycan in the matrix. **B** Representative histograms of UC-MSC surface markers, cell viability and apoptosis/necrosis. The isotype control is shown as a red line histogram. **C** UC-MSC karyogram after cell expansion. Normal karyotype: 46, XX. **D** Representative histograms from the lymphocyte inhibition assay. MSCs were cultivated with PHA stimulated CD3 + lymphocytes labeled with CFSE. (a) CD3 + lymphocytes not labeled with CFSE; (b) CFSE-labeled CD3 + lymphocytes; (c) CD3 + lymphocytes labeled with CFSE and stimulated with PHA (1 µg/µL); (d) MSCs were cultivated with CD3 + lymphocytes labeled with CFSE 1:10; (e) MSCs were cultivated with CD3 + lymphocytes labeled with CFSE 1:5; (f) MSCs were cultivated with CD3 + lymphocytes labeled with CFSE in a 1:2 ratio

The ATP infused into the patients was negative for microbiological tests, and no clonal chromosomal abnormalities were observed. For each infusion, the average cell viability was 96.6 ± 0.01, 95.4 ± 0.03 and 95.5 ± 0.02, respectively. Cell characterization was performed following the criteria defined by International Society for Cellular Therapy (ISCT) Guidelines (Additional file 1: Table S1). UC-MSCs show the potential to differentiate into osteoblasts, adipocytes and chondroblasts, and the immunomodulation potential is higher than 50% (Fig. 2). The results from all evaluations were in conformation with those established by CCT/PUCPR.

### Patient baseline characteristics and study population

A total of 17 patients were included in this study from 12 June to 13 July 2020. After randomization, 11 patients were included in the UC-MSC group, and 6 patients in the placebo group. In the UC-MSC group, one patient was excluded on the twelfth day because she did not undergo follow-up after cell infusion. At enrollment, all patients had ARDS and were in IMV in critical condition rated 6–7 on the WHO scale.

The average age of the UC-MSC group was 53 ± 15.3 years, while the average age of the placebo group was 61.7 ± 9.7 years. The baseline symptoms were fever, cough, nausea or vomiting, diarrhea, loss of taste or smell, shortness of breath, disorientation and confusion. No differences were observed when comparing the interval between symptom onset and hospital admission or the interval between symptom onset and first cell injection. Two patients in the UC-MSC group had no basic chronic diseases, and all other patients had comorbidities when they were admitted to the hospital, such as diabetes, hypertension, kidney disease, chronic obstructive pulmonary disease, schizophrenia and obesity. The patients received standard treatment with anticoagulants, steroids and antibiotics if there was evidence of bacteriological infection. Two patients from the UC-MSC group were treated with antiviral drugs as a concomitant treatment. Patient baseline characteristics are demonstrated in Table 1.

**Table 1:** Baseline characteristics

Characteristics		UC-MSC (*n* = 11)	Placebo (*n* = 6)
Age (years)	Mean ± SD	53 ± 15.3	61.7 ± 9.7
Gender, *n* (%)	Female	3 (27.2)	2 (33.3)
	Male	8 (72.7)	4 (66.6)
Symptoms, *n* (%)	Fever	8 (72.7)	5 (83.3)
	Cough	9 (81.8)	4 (66.6)
	Nausea or vomiting	3 (27.3)	2 (33.3)
	Diarrhea	4 (36.4)	0 (0)
	Loss of taste or smell	4 (36.4)	3 (50)
	Shortness of breath	9 (81.8)	3 (50)
	Disorientation and confusion	2 (18.2)	0 (0)
*Oxigenation index (PaO_2_/FiO_2_) at enrollment, *n* (%)	Mild	4 (36.4)	5 (83.3)
	(200˂PaO_2_/FiO_2_ ≤ 300 mmHg)		
	Moderate	6 (54.5)	0 (0)
	(100˂PaO_2_/FiO_2_ ≤ 200 mmHg)		
	Severe	1 (9.1)	1 (16.6)
	(PaO_2_/FiO_2_ ≤ 100 mmHg)		
Interval between symptoms onset and hospital admission (days)	Mean ± SD	6.8 ± 3.2	8 ± 1.6
Interval between symptoms onset and first cell injection (days)	Mean ± SD	10.7 ± 3.9	12.1 ± 2.2
Comorbidities, *n* (%)	Diabetes	4 (36.4)	3 (50)
	Hypertension	6 (54.5)	3 (50)
	Kidney disease	1 (9.1)	0 (0)
	Chronic obstructive pulmonary	0 (0)	1 (16.7)
	Disease		
	Schizophrenia	1 (9.1)	0 (0)
	Obesity (BMI > 30)	6 (54.5)	3 (50)
Concomitant treatment, *n* (%)	Anticoagulant	11 (100)	6 (100)
	Steroids	11 (100)	6 (100)
	Antibiotics	2 (18.2)	1 (16.7)
	Antiviral drugs	2 (18.2)	0 (0)

*n*, number; PaO_2_, arterial oxygen partial pressure; FiO_2_, oxygen absorption concentration; UC-MSC, umbilical cord mesenchymal stromal cell; BMI, body mass index

Laboratory tests, such as blood count and serologic, biochemical and cell subpopulations, were evaluated at baseline (Table 2). All these parameters were considered response variables in the statistical analysis (Additional file 2: Tables S2, Additional file 3: Table S3).

**Table 2:** Patient laboratory findings

Laboratory tests		UC-MSC (*n* = 11)	Placebo (*n* = 6)	*p* value
Blood count	Total lymphocyte (µL)	737 ± 299.55	1652.33 ± 2032.80	0.01
	Leukocytes (µL)	10,959.73 ± 4591.77	9605 ± 3998.52	n.s
	Neutrophils (µL)	9899.73 ± 4354.47	8266 ± 3671.02	n.s
	Hemoglobin (µL)	13.35 ± 1.69	12.68 ± 2.56	n.s
	Hematocrit (%)	41 ± 5	38 ± 7	n.s
	Platelets (µL)	271,690.91 ± 75,889.73	296,500 ± 54,013.89	n.s
Serologic	D-Dimer (mg/L)	7.75 ± 10.81	6.45 ± 4.83	n.s
	C-Reactive protein (mg/dL)	38 ± 81	9 ± 5	n.s
	Ferritin (ng/mL)	2760.53 ± 3167.67	1600.38 ± 1258.86	n.s
	Troponin I (pg/mL)	218 ± 655	132 ± 209	n.s
Biochemical	Creatinin (mg/dL)	2.07 ± 1.71	1.92 ± 1.84	n.s
	ALT (µ/L)	49 ± 40	29 ± 14	n.s
	AST (µ/L)	60 ± 33	45 ± 19	n.s
	Total Bilirubin (mg/dL)	0.84 ± 1.41	0.33 ± 0.12	n.s
	Direct bilirubin (mg/dL)	0.59 ± 1.17	0.18 ± 0.06	n.s
	Indirect bilirubin (mg/dL)	0.24 ± 0.28	0.15 ± 0.10	n.s
Main lymphocytes subgroups	TCD3	242.17 ± 176.44	471.90 ± 205.33	0.02
	TCD4	195.120 ± 94.84	346.06 ± 194.50	0.01
	TCD8	105.83 ± 85.91	107.06 ± 41.42	n.s
	B	90.47 ± 53.31	143.56 ± 146.16	n.s
	NK	56.54 ± 35.54	41.92 ± 27.21	n.s
	Treg	11.26 ± 6.20	21.57 ± 6.66	n.s
	Plasmablasts	17.04 ± 17.01	14.94 ± 7.88	n.s

ALT, alanine aminotransferase; AST, aspartate aminotransferase; NK, natural killer; n.s, not significant; Treg, CD4 regulatory T lymphocytes; UC-MSC, umbilical cord mesenchymal stromal cell

### The primary safety outcome

Safety was evaluated through AEs observed within 24 h after each infusion, including clinical examinations and measurement of vital signs. No serious complications associated with UC-MSC infusion were observed. One patient from the UC-MSC group had transient hypotension after the first infusion. In the placebo group, one patient had tachycardia immediately after the first infusion; however, the alveolar recruitment maneuver was performed by physiotherapy, and it is not possible to be sure that this would not be the cause of the observed tachycardia. There were no clinical repercussions for the patient and no need for intervention. No AEs were observed in the second and third cell infusions. Critically ill patients with severe COVID-19 showed no immediate deaths or acute anaphilatic shock after UC-MSC infusion. The other patients showed stable vital signs after the treatment. Investigators conducted assessments for the presence of any AEs from enrollment throughout the study.

In this study, five patients from the UC-MSC group and one patient from the placebo group (35% of the patients) passed away, although no significant difference was observed between the groups. Five patients were male and one female, and their ages ranged from 41 to 78 years. None of the deaths seemed to be related to UC-MSC infusion. The cause of death of five patients was secondary to bacterial septic shock, and one patient died secondary to ARDS and multiorgan dysfunction syndrome. There was no association of mortality and elderly patients. Five of the six patients had comorbidities such as obesity, diabetes, hypertension and schizophrenia, which was not associated with mortality. However, patients who presented dialysis kidney dysfunction during the course of the disease had higher associated mortality (*p* = 0.029). Table 3 shows the details of death.

**Table 3:** Survival status of COVID‐19 patients

Patient	Interval between first cell infusion and death (days)	Study group	Oxygenation PaO_2_/FiO_2_	Comorbidities	Cause of deaths
1	8	UC-MSC	173 mmHg—moderate	Hypertension, obesity	Multiorgan dysfunction syndrome
6	8	UC-MSC	175 mmHg—moderate	Diabetes, hypertension	Bacterial septic shock
8	20	UC-MSC	250 mmHg—mild	Hypertension	Bacterial septic shock
10	17	UC-MSC	180 mmHg—moderate	None	Bacterial septic shock
15	38	Placebo	99 mmHg—severe	Diabetes	Bacterial septic shock
17	23	UC-MSC	96 mmHg—severe	Diabetes, schizophrenia	Bacterial septic shock

PaO_2_, arterial oxygen partial pressure; FiO_2_, oxygen absorption concentration; UC-MSC, umbilical cord mesenchymal stromal cell

### The efficacy outcome after four month follow-up

#### HCoV-19 nucleic acid detection

Viral load was performed at baseline and after cell therapy on days 2, 4, 6 and 14, and blood count, serologic tests, biochemistry, cell subpopulation analysis and inflammatory cytokines were performed at all times already mentioned, including 2 and 4 months.

**Fig. 3:**
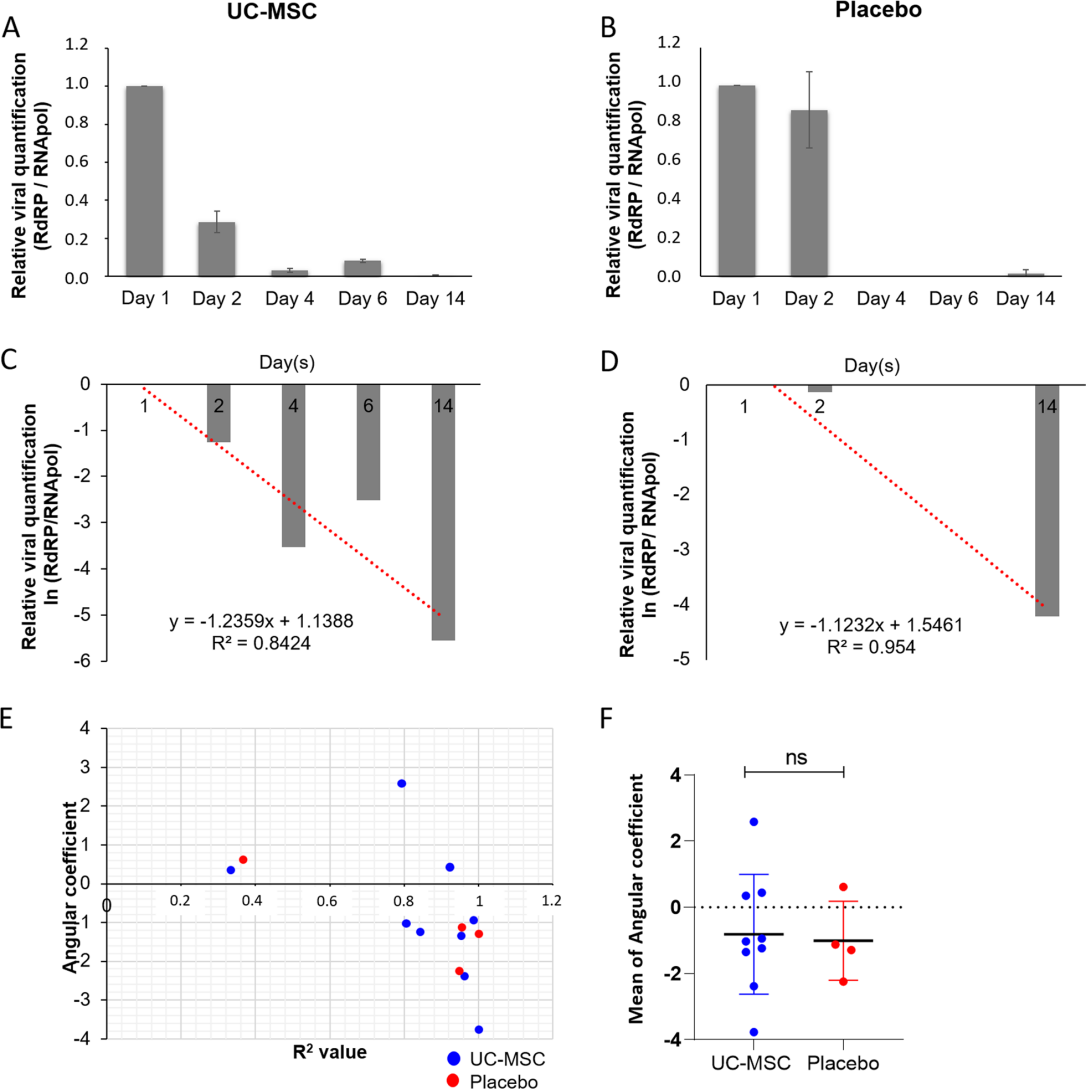
Viral load from day 1 (baseline) to 14 days after UC-MSC treatment. **A**–**D** Graphs representing relative viral quantification. Viral load was determined based on the relative expression of the viral gene RdRP in relation to the human POLR2B gene (normalizer). Viral gene expression gradually decreased in patient samples from day 1 (baseline) to day 14 (**A**, **B**). **C**, **D** After linearizing the data by the natural logarithm (ln (*x*)) and obtaining the linear equation, the angular coefficient of the viral load line (slope) and the coefficient of determination (*R*^2^) were established. **C** UC-MSC and **D** Placebo. The angular coefficient (slope) and *R*^2^ of each patient are plotted in (**E**). Average of the angular coefficient of each group (**F**). Mean with SEM; Student's unpaired *t* test analysis. UC-MSC, umbilical cord mesenchymal stromal cell; ns, not significant

Quantification of viral load was assessed in patient samples at baseline and 2, 4, 6 and 14 days after infusion with UC-MSCs or placebo (Fig. 3). The angular coefficient (slope) and *R*^2^ value were established for each patient. Most patients had an angular coefficient below zero, which means that the viral load decreased over time (Fig. 3C–E). Only two patients (1 UC-MSC and 1 placebo) did not obtain strong *R*^2^ values (< 0.4, Fig. 3E). The data showed no significant difference in viral load, as determined by the angular coefficient, between the UC-MSC and placebo groups (Fig. 3F). In this study, in both groups, there was a reduction in viral load over time, without significant differences.

### Laboratory assessments

*Analysis of* coagulation parameters.

**Fig. 4:**
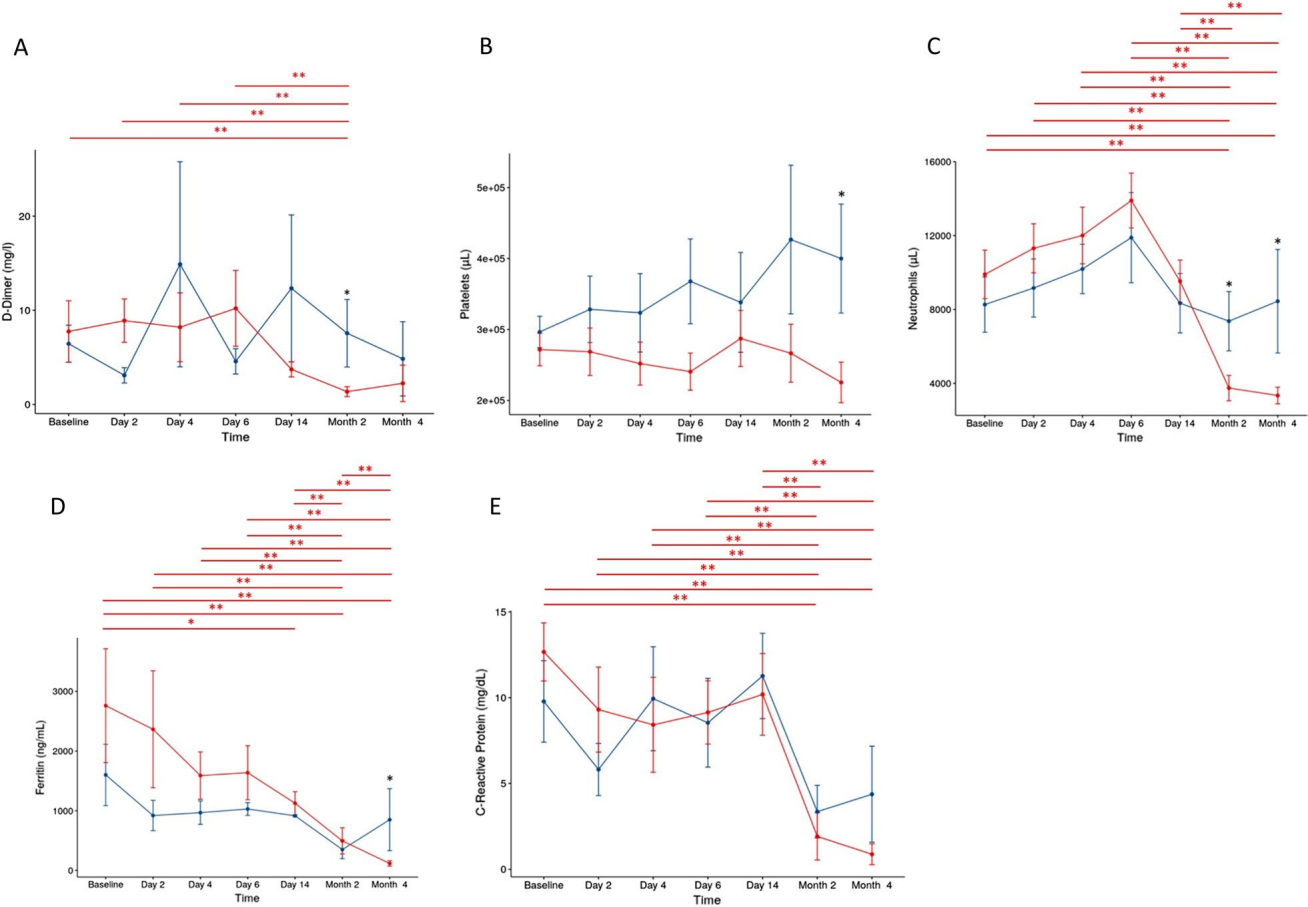
Coagulation parameters and inflammatory markers. Comparison between UC-MSC (red line) and placebo (blue line) groups over time. The bars show standard deviations (SD). *p ≤ 0.05 for comparison between groups (black asterisk). Statistical differences inside groups, longitudinal follow-up, were depicted in the graphs (UC-MSC—red line, placebo—blue line). *p ≤ 0.05 and **p ≤ 0.01 for longitudinal follow-up (red and blue asterisks). **A** D-dimer, **B** Platelets, **C** Neutrophils, **D** Ferritin and **E** C-Reactive Protein. UC-MSCs, umbilical cord mesenchymal stromal cells

Coagulation markers were also evaluated, including D-dimer, platelets and neutrophils. With respect to D-dimer, both groups presented values above the reference in all evaluations. However, in the UC-MSC group, a decrease in D-dimer values was observed in the second month compared to baseline (*p* = 0.01). At 2 months, values in the UC-MSC group were very close to the reference and significantly lower than the value in the placebo (UC-MSC vs placebo, *p* = 0.01). In the UC-MSC group, there was a decrease in the number of platelets in the comparison between baseline and 2 months (*p* = 0.01) and 4 months (*p* = 0.01). In the second and fourth months, platelets in the UC-MSC group were within the reference range, whereas in the placebo group, they were out of the reference range. In the fourth month, there was a significant difference, with higher levels in the placebo group (*p* = 0.01). A difference in the number of neutrophils in both groups was also observed. The UC-MSC group had a lower number of neutrophils than the placebo group in the second month (*p* = 0.03) and fourth month (*p* = 0.01) after treatment. These results demonstrate that two months after cell infusion, there was a decrease in coagulation markers that could reduce the risk of thrombus formation compared to the placebo group (Fig. 4).

#### Analysis of inflammatory markers

To determine the patients' inflammatory status, ferritin, C-reactive protein and cytokine levels were analyzed. Ferritin values in the UC-MSC group were higher at baseline than at day 14 (*p* = 0.03), 2 months (*p* = 0.01) and 4 months (*p* = 0.01). In the fourth month, there was a marked and statistically significant decrease in ferritin values and a return to normal levels. In the placebo group, the levels were always higher than the reference ranges. In the fourth month, there was an increase in the placebo group, opposite to the UC-MSC group (*p* = 0.01). C-reactive protein, which is the main inflammatory marker in COVID-19 patients, showed a decrease in the comparison between baseline at 2 months (*p* = 0.01) and baseline at 4 months (*p* = 0.01) in the UC-MSC group. In the second month, the values were within normal levels, while in the placebo group, although the values were lower than those in the UC-MSC group, there were no differences over time, always maintaining levels above the reference (Fig. 4).

**Fig. 5:**
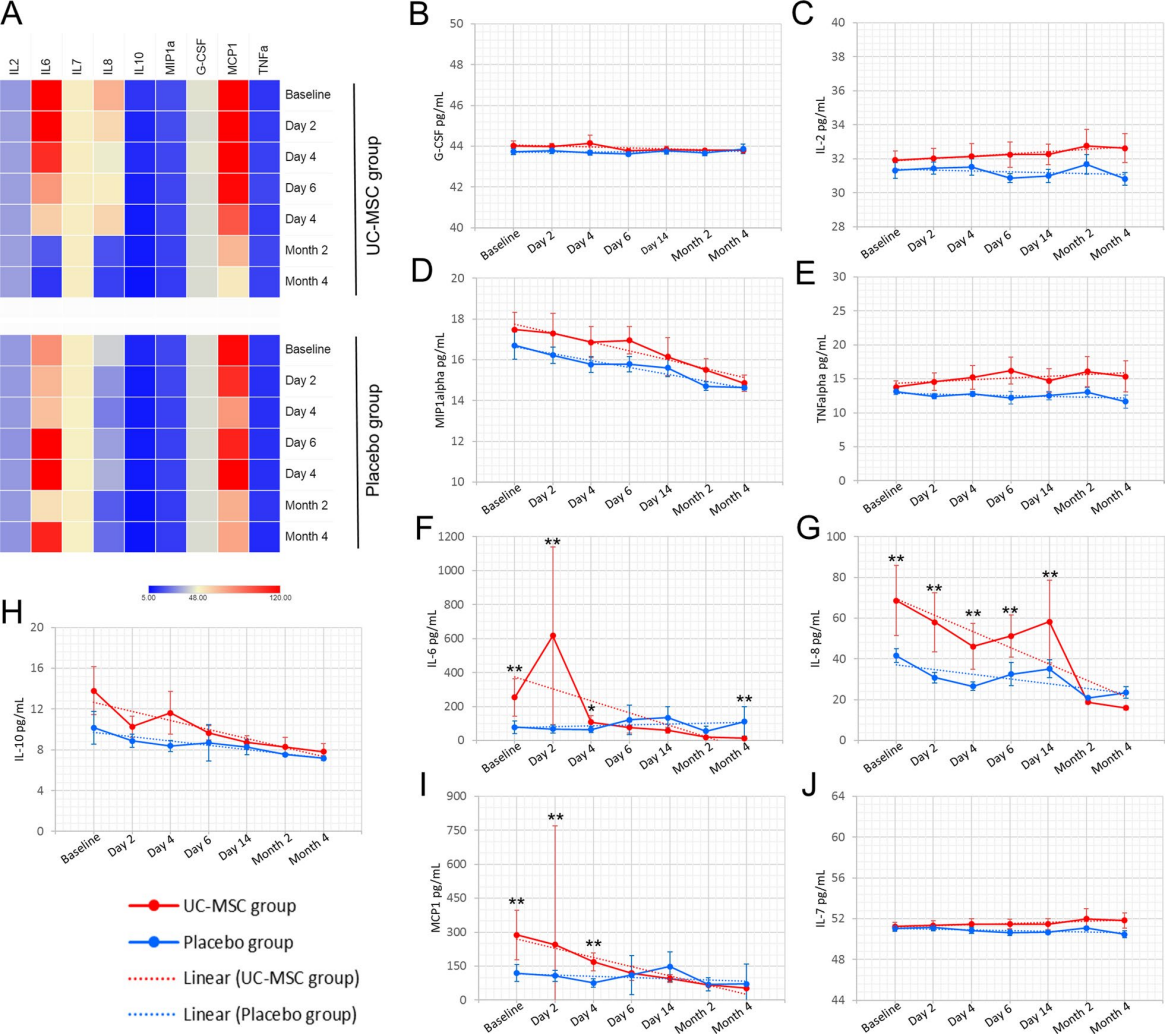
Profile of plasma cytokines, chemokines and growth factors in the patients during different clinical stages. Comparison between groups (UC-MSCs vs Placebo) and evaluated time points (baseline, days 2, 4, 6, 14 and 2 and 4 months). **A** Heatmap jointly comparing all analytes evaluated over the treatment time, either with cell therapy (UC-MSCs group) or placebo. Graphs show the results of some analytes separately, comparing the UC-MSC (red line) and placebo (blue line) groups over time. The bars show standard deviations (SD), and the broken line is the trend line fitted to the data. **B** Granulocyte–Macrophage Colony-Stimulating Factor (GM‐CSF); **C** Interleukin 2 (IL-2); **D** Macrophage Inflammatory Protein 1-Alpha (MIP1a/CCL3); **E** Tumor Necrosis Factor (TNF); **F** Interleukin 6 (IL-6); **G** Interleukin 8 (IL-8); **H** Interleukin 10 (IL-10); **I** Monocyte chemoattractant protein-1 (MCP1-CCL2); **J** Interleukin 7 (IL-7). Abbreviations: UC-MSCs, Umbilical Cord Mesenchymal Stromal Cells. *p ≤ 0.04 and **p ≤ 0.02 for comparison between groups. Statistical differences inside groups, longitudinal follow-up, were not depicted in the graphs but indicated in the main text

Regarding the cytokines IL-2, MIP1a-CCL3, G-CSF and TNFα assays, there were no differences between and within the time points of each group (Fig. 5). Conversely, IL-6 levels in the UC-MSC group showed differences between baseline and the fourteen day (*p* = 0.02), second month (*p* = 0.01) and fourth month (*p* = 0.01). From the fourteen day onwards, IL-6 levels decreased significantly (fourteen day vs second month, *p* = 0.01; fourteen day vs fourth month, *p* = 0.01). During this same period, in the placebo group, the levels remained high, with no differences between evaluation time points. In the comparison between groups, at baseline (*p* = 0.01), day 2 (*p* = 0.01) and day 4 (*p* = 0.04), IL-6 values were higher in the UC-MSC group than in the placebo group. However, at month 4, there was a significant decrease in the UC-MSC group and an increase in the placebo group (*p* = 0.01). The levels of IL-8 in the UC-MSC group until the fourteen day were always higher than those in the placebo group. In both groups, there was a large reduction in values at 2 and 4 months (UC-MSC group, baseline vs 2 months, *p* = 0.01; baseline vs 4 months, *p* = 0.01; placebo group, baseline vs 2 months, *p* = 0.01, baseline vs 4 months, *p* = 0.01). The UC-MSC group showed a decrease in MCP1 levels, with differences between baseline and the fourteen day (*p* = 0.01), 2 months (*p* = 0.01) and 4 months (*p* = 0.01). Comparing groups, this cytokine level was higher in the UC-MSC group than in the placebo group at baseline (*p* = 0.01), 2 days (*p* = 0.01) and 4 days (*p* = 0.01). However, from the sixth day to the fourth month, there was a decrease, with no differences in relation to the placebo group. Regarding the cytokines IL-6, IL-8 and MCP1-CCL2, all had higher levels in the UC-MSC group than in the placebo group until the fourth day. After this period, the levels decreased and were lower than those in the placebo group in the fourth month, suggesting that MSCs were effective in decreasing inflammation. The level of IL-7, which is a pleiotropic cytokine essential for lymphocyte survival and expansion, showed a decrease in the fourth month in the placebo group (baseline vs 4 months, *p* = 0.02), indicating a worsening for patients in this group (Fig. 5).

#### Analysis of cell subpopulation

**Fig. 6:**
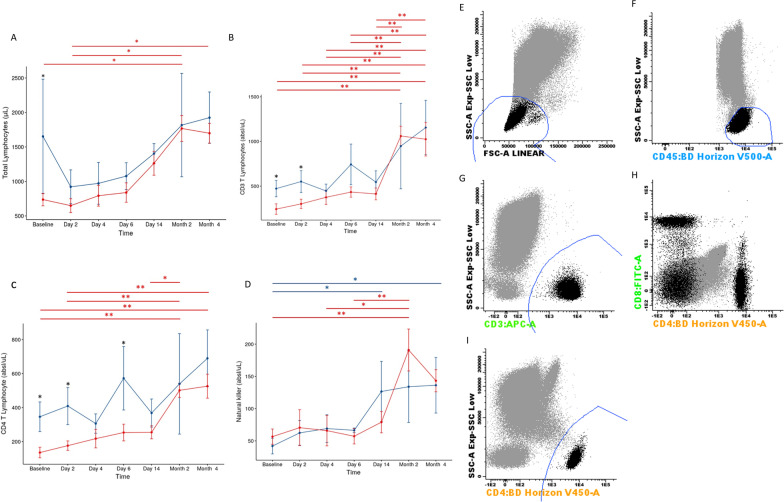
Analysis of cell subpopulation. **A** Total lymphocytes, **B** CD3 T lymphocytes, **C** CD4 T lymphocytes, **D** Natural killer. Comparison between UC-MSC (red line) and placebo (blue line) groups over time. The bars show standard deviations (SD). *p ≤ 0.05 for comparison between groups (black asterisk). Statistical differences inside groups, longitudinal follow-up, were depicted in the graphs (UC-MSC—red line, placebo—blue line). *p ≤ 0.05 and **p ≤ 0.01 for longitudinal follow-up (red and blue asterisks). Flow cytometry strategy for lymphocyte analysis, total lympohocytes, CD3 T, CD8 T and CD4 T lymphocytes. **E** Lymphocyte gate FSC/SSC low; **F** Strong CD45 +  + ; **G** CD3 positive; **H** CD4/CD8 dotplot; **I** CD4 positive. UC-MSCs, umbilical cord mesenchymal stromal cells; SSC, side scatter; FSC, forward scatter

In this trial, the main cell subpopulations were evaluated by flow cytometry. The total lymphocyte count at baseline was below the reference range in the UC-MSC group and significantly lower than that in the placebo group (*p* = 0.01). From the sixth day, there was an increase in the lymphocyte count at UC-MSC group, with a return to the normal range and a difference between baseline and 2 months (*p* = 0.04). In the placebo group, at all evaluation times, values were within reference. The numbers of CD3 T and CD4 T lymphocytes were also lower in the UC-MSC group than in the placebo group at baseline (*p* = 0.02 and *p* = 0.01, respectively) and on the second day after infusion (*p* = 0.04 and *p* = 0.01, respectively), with no significant differences in the other evaluations. These results indicate that the placebo group was in better condition than the UC-MSC group at the beginning of the study. From the second month, an increase in the absolute CD3 T lymphocyte values was observed, with differences between baseline and 2 months (*p* = 0.01) and 4 months (*p* = 0.01) in the UC-MSC group. The same was observed in relation to CD4 T lymphocytes (baseline vs 2 months, *p* = 0.01; baseline vs 4 months, *p* = 0.01). In the placebo group, there were no differences over time. Values of NK cells increased significantly when comparing baseline and 2 months (*p* = 0.01) in the UC-MSC group. These data indicate that lymphopenia, which has important prognostic potential and is present in patients who need intensive treatment, was more common in patients in the UC-MSC group, and after cell infusion, there was an improvement in immune system function (Fig. 6).

Even with a small number of individuals per group, the results presented thus far strongly suggest that treatments with MSCs significantly improve several of the patients' functional and inflammatory parameters.

### The efficacy outcome: hepatic, cardiac, kidney and pulmonary sequelae

**Fig. 7:**
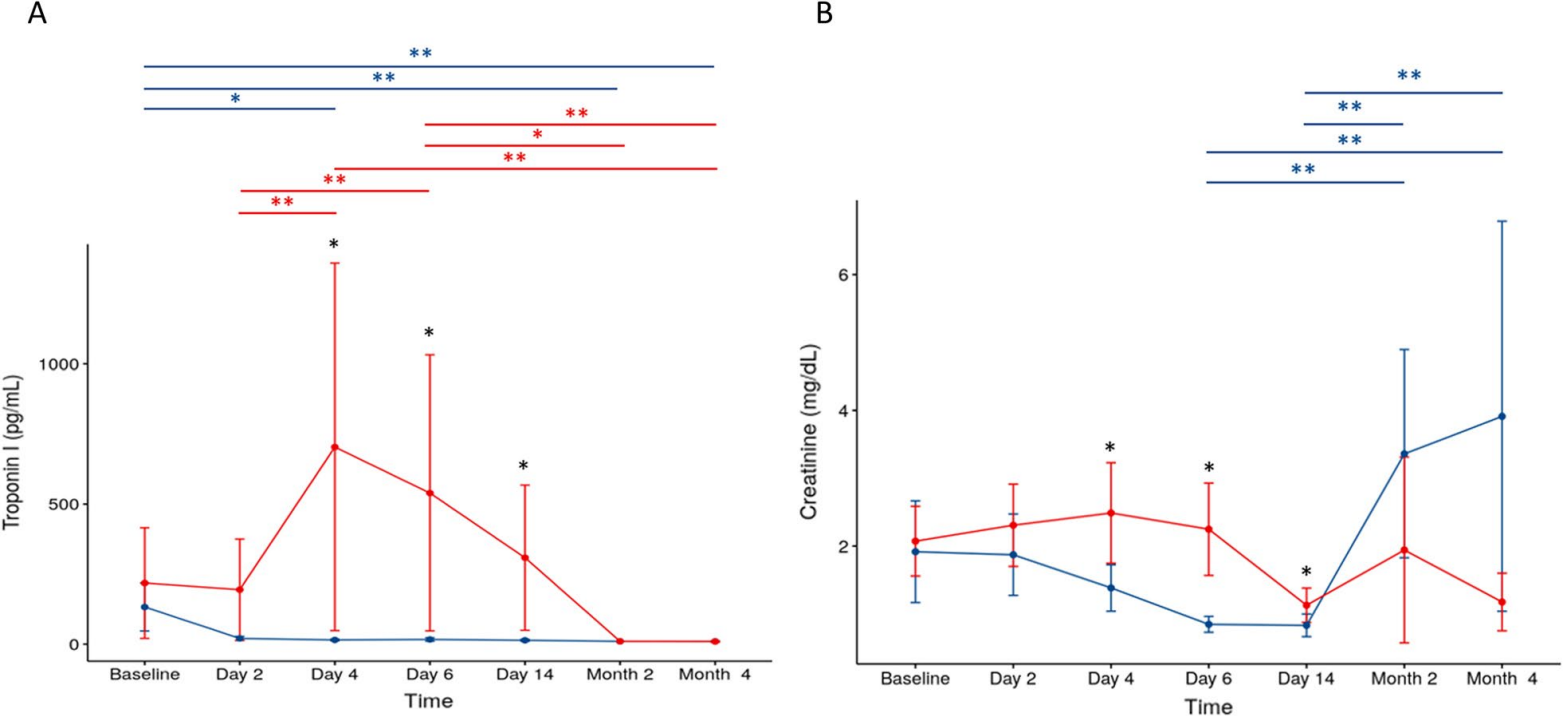
Cardiac and kidney function markers. Comparison between UC-MSC (red line) and placebo (blue line) groups over time. The bars show standard deviations (SD). *p ≤ 0.05 for comparison between groups (black asterisk). Statistical differences inside groups, longitudinal follow-up, were depicted in the graphs (UC-MSC—red line, placebo—blue line). *p ≤ 0.05 and **p ≤ 0.01 for longitudinal follow-up (red and blue asterisks). **A** Troponin I and **B** Creatinine. UC-MSCs, umbilical cord mesenchymal stromal cells

Months after COVID-19 infection, patients could still present some persistent symptoms that need to be monitored. Some biochemical tests to evaluate liver function, such as bilirubin, alanine aminotransferase (AST) and aspartate aminotransferase (ALT), showed no differences between groups. Regarding the function of the kidneys and heart, the levels of troponin I and creatinine were analyzed. Troponin I showed decreased levels in both groups. Values were within the normal range at the second and fourth months after treatment (placebo group, baseline vs 2 months, *p* = 0.01; baseline vs 4 months, *p* = 0.01/UC-MSC group, 6 days vs 2 months, *p* = 0.04; 6 days vs 4 months, *p* = 0.01). Creatinine values indicative of renal function in the UC-MSC group were increased compared to those in the placebo group on days 4 (*p* = 0.03), 6 (*p* = 0.01) and 14 (*p* = 0.02). On day 14, after three cell infusions, values were within the reference range, showing an improvement in renal function. In the second and fourth months, the UC-MSC group remained within the reference range, while there was an increase in levels in the placebo group above normal values, but there was no significant difference between groups (Fig. 7).

**Fig. 8:**
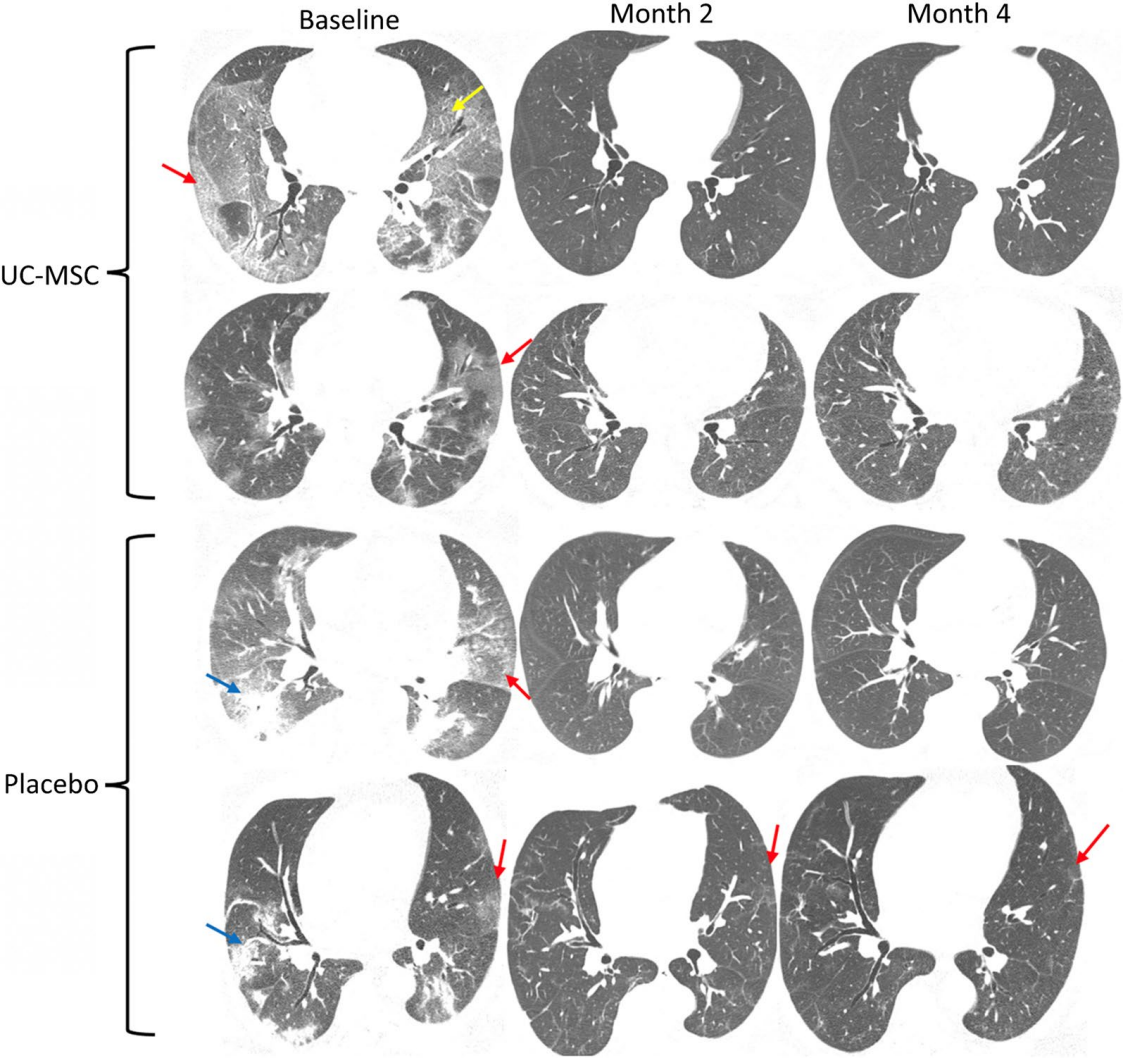
Representative images from chest CT at the level of the lower lobes in patients from the treatment and control groups. Red arrows point to ground-glass opacification, blue arrows to peripheral consolidations, and the yellow arrow to crazy-paving, all of which are typical features of COVID-19. It is possible to note the higher degree of clearance in patients from the treatment group (first line) compared to the control group (last line). UC-MSC, umbilical cord mesenchymal stromal cell

In this trial, chest CT was used to detect lung damage in COVID-19 patients. All patients suffered from serious pulmonary damage and needed oxygen inhalation support during the course of disease. CT imaging results revealed bilateral, multilobular involvement as well as segmental consolidation and characteristics of pneumonia. Concerning chest CT abnormalities, there was no significant difference between groups (Additional file 4: Table S4). However, there was a reduction in the extension of opacities related to COVID-19 in chest CT scans for both groups (Additional file 2: Table S2). Visually, there was a higher degree of clearance in patients from the UC-MSC group than in the placebo group, with statistical analysis showing a significant difference in the degree of opacification in those patients when comparing baseline and 4 months (*p* = 0.01) and 14 days and 4 months (*p* = 0.01). Patients with pulmonary fibrosis were not observed (Fig. 8).

## Discussion

Severe SARS-CoV-2 infection induces a cytokine storm, leading to ARDS and MODS, which are very serious health conditions. Those characteristics of COVID-19 make disease control challenging if using a single strategy. MSC properties allow the systemic distribution of positive immunomodulatory and regenerative effects throughout the body, thereby ensuring a systemic effect in addition to local modulation [70]. To our knowledge, this is one of the few reports that presents the longest follow-up after MSC treatment in COVID-19 patients. This trial was conducted during the early stages of the COVID-19 outbreak and proposed UC-MSC infusion as an adjunctive therapy in the recovery and postacute sequelae reduction caused by COVID-19. The results indicate tolerability and safety and suggest the efficacy of UC-MSC infusion in critically ill patients.

In the present study, fresh allogeneic UC-MSCs were used in all infusions. These cells were infused within 72 h after the patient's inclusion in the study. Some studies show that MSCs need to restart their metabolism and other biochemical processes before infusion, recovered by a 24–72 h subculture, to restore their functionality [71, 72]. Therefore, the use of fresh cells in this study may have been important for the results observed.

Sample randomization was conducted in the preliminary experimental design to protect against imbalance in biasing caused by enrollment. However, with small numbers of patients, there is still potential for imbalance. In this study, some differences between groups were observed at baseline, demonstrating that the UC-MSC group seemed to be more compromised than the placebo group.

Concerning baseline characteristics, all patients received standard treatment with steroids to inhibit the inflammatory response, specially indicated for those requiring respiratory support [73]. Besides, treatment with glucocorticoids and heparin reduce neutrophil extracellular trap (NET) formation [74, 75], as it has been shown to have therapeutic value in COVID-19 treatment [76]. In this research, two patients in the UC-MSC group had no previous chronic diseases, while all other patients had comorbidities. This is in line with studies that show that the presence of underlying conditions such as cardiovascular disease, chronic pulmonary disease and diabetes are risk factors that will require critical care [77].

The interval between the first infusion and hospital discharge was similar between groups, showing that MSCs do not accelerate patient recovery. Similar results were observed by Adas et al. [51], when compared the control and experimental groups of critically ill patients. The average length of stay was 45 days in the control group and 47 days for the placebo group, with no significant differences.

Safety evaluation after three IV UC-MSC infusions, showed only mild AEs and they were unrelated to cell infusion, indicating safety with no life-threatening complications. Safety was shown in other studies that also performed MSC-based interventions. They did not noticed any acute infusion-related issues, allergic reaction, delayed hypersensitivity or secondary infections in the patients either [40, 46–49, 55, 58, 78, 79]. According to Saleh et al. [54], the optimal time for cell infusion is the second week of the disease, namely, the second phase, where there is hyperinflammation beginning on days 7–15. Zhu et al. [57] also suggested that MSCs can improve the outcome of patients with severe/critical symptoms more significantly than common/mild patients. In this trial, only critically ill patients were included, and the average interval between symptom onset and first cell injection was 10.7 days for the UC-MSC group and 12.1 days for the placebo group. Possibly due to a combination of suitable patient and the ideal time points for patient treatment, an overall patient benefit after IV UC-MSC infusion was noticed.

The mortality rate in this study was 35%, of which 83.3% were men. The most common cause of death was secondary bacterial infection. A similar study carried out in Brazil showed that in the same period, the mortality rate in ICU patients in the South region was 53% [80]. Hashemian et al. [53] studied critically ill patients with severe hypoxemia who required MV and observed a 45% mortality rate—most of which had signs of multiorgan failure or sepsis, and died 5–19 days after the first infusion. Also Dilogo et al. [52] showed that the mortality rate was 65% in a group of intubated critically ill patients with COVID-19 in the ICU, and higher mortality was associated with patients who had two or more comorbidities. Another trial with critically ill patients was conducted by Adas et al. [51], and they observed 33% mortality in patients who received MSC therapy. The most common cause of death was secondary infections due to bacteria, followed by myocardial infarction and thromboembolism. All these studies reinforce that critically ill COVID-19 patients had little chance of survival and MSC treatment may increase the survival rate. Gender was also shown to be related to mortality. The ACE2 gene localizes on the X chromosome, and ACE2 levels in the blood are higher in males than in females as well as in patients with diabetes or cardiovascular disease [81–84].

In our research, patients who presented dialysis kidney dysfunction had higher associated mortality. The incidence of acute kidney injury (AKI) secondary to COVID-19 is high [85] and is strongly associated with high mortality among hospitalized COVID-19 [86–88]. Ghonimi et al. [89] reported a strong association between death related to COVID-19 infection in dialysis patients, which was also observed in the study of Costa et al. [90], showing that COVID-19 patients with AKI who need dialysis had worse outcomes. Lino et al. [91] also showed that a worse prognosis is frequently associated with a more rapid evolution to intensive and respiratory care or even dialysis.

The systemic dissemination of the virus or viral components is associated with the severity of COVID-19 and with a number of parameters indicating the presence of a dysregulated response to the infection [92]. To evaluate the impact of the extrapulmonary dissemination of viral material on disease severity and on the host response to the infection, it was verified the presence of genomic material of the virus in plasma. In this study, in both groups, 14 days after infusion, there was a reduction in viral load over time, without significant differences. This is in line with Lanzoni et al. [47], who showed no differences between the UC-MSC treatment and the control group. According to the authors, UC-MSC treatment seems to be more closely associated with a decrease in inflammatory cytokines rather than a change in viral load. Leng et al. [40] also observed that critically severe patients became negative for hCoV-19 nucleic acid 13 days after transplantation.

Neutrophils and the imbalance between NET formation and degradation play a central role in the pathophysiology of inflammation, coagulopathy, organ damage and immunothrombosis [93, 94]. Clinical studies have found that the number of neutrophils in the bronchoalveolar lavage fluid of ARDS patients is correlated with the severity of COVID-19 and the cytokine storm [95]. Circulating platelets bind neutrophils and may result in NET formation in the pulmonary and renal microcirculation [96], thereby contributing to immunothrombosis in patients with COVID-19 [93]. Zhu et al. [57] found that MSC treatment can reduce plasma NET-DNA levels in COVID-19 patients. In this research, in the second month after UC-MSCs treatment, there was a reduction in the number of neutrophils to the reference range. Platelet values were at the reference range in UC-MSC group, while in the placebo group, in the second and fourth months, the number increased above the reference. The results indicate a lower risk of thrombosis in the UC-MSC group. In the evaluation of D-dimer, a biomarker for thrombotic disorders and a potential indicator for prognosis in COVID-19 patients [97–99], both groups presented D-dimer values above the reference in all evaluations. However, in the UC-MSC group, a decrease in D-dimer values was observed in the second month suggesting that UC-MSC group might decrease the risk of thrombosis formation in critically ill patients with COVID-19.

In the acute phase reaction of an inflammatory process, there is a variation in the concentrations of various plasma proteins, including C-reactive protein and ferritin. They are important biomarkers of inflammation in the context of COVID-19 progression because they are predictive of in-hospital [100, 101]. Patients with the highest ferritin levels also presented significantly higher levels of C-reactive protein and serum creatinine [91]. In the present trial, analysis of inflammatory markers showed that C-reactive protein and ferritin values, in the UC-MSC group, decreased in the second and fourth months compared with baseline. In the placebo group, the levels were always higher than the reference ranges. Those results are in accordance with studies that show that the inflammatory biomarkers were increased in COVID-19 patients [102, 103].

The CRS storm, present in critically ill COVID-19 patients, occurs due to the combination of a defective (or delayed) first line of defense, followed by persistent hypercytokinemia and a dysfunctional T cell response [104–107]. The clinical manifestation is the sharp rise of a large number of cytokines within a short time frame. Liu et al. [108] identified that serum levels of IL-6 (> 32.1 pg/mL), one of the mediators of hyperinflammation, have a significant correlation with the severity of COVID-19 and can be used to predict disease risk. In the present study, increased levels of IL-6 were observed in both groups. In the placebo group, the levels remained high at the second (mean 45.97 pg/mL) and fourth month (100.97 pg/mL). Conversely, there was a significant decrease from day 14 to the fourth month in the UC-MSC group. Besides, it was observed a decreased in IL-8 and MCP-1 levels after UC-MSCs infusion. IL-8, considered a biomarker for COVID-19 disease prognosis [109, 110], in this study, was decreased at 2 and 4 months, in both groups. This same result was obtained by Li et al. [110], who showed that serum levels of IL-8 correlated to the overall clinical disease scores at different stages of the same COVID-19 patients. Hence, higher levels in the UC-MSC group compared to placebo group until the day 14, enforced that the patients in this group had a worse prognosis than those in the placebo group. The MCP-1-CCL2 chemokine level in the UC-MSC group, until the fourth day, was higher than that in the placebo group, decreasing from the day fourteen to the fourth month, reaching levels with no differences in relation to the placebo group. This chemokine attracts or enhances the expression of other inflammatory factors/cells, is associated with the severity of COVID-19 disease and can be related to the risk of death in COVID-19 patients [111]. Cytokine results reveal that there was decreased inflammation and clinical improvement in the patients treated with MSCs.

In the placebo group, the IL-7 level was decreased in the fourth month, with a significant difference compared to baseline. IL-7 promotes lymphocyte expansion and possibly reverses T cell exhaustion and may be useful in restoring immune system homeostasis [112]. This cytokine exerts antiapoptotic properties and induces potent proliferation of naive and memory T cells, leading to replenishment of circulating CD4 T and CD8 T lymphocytes [113, 114]. Most likely, for this reason, a recovery in the CD3 T, CD4 T and NK lymphocyte numbers was not observed in the placebo group at different evaluation times.

Studies that have shown that all subsets of lymphocytes were decreased in COVID-19 patients [115–118] and that T cells exhibit elevated exhaustion levels and reduced functional diversity [119, 120]. In the present study, lymphopenia was also observed, due to the severity of the disease, although the number of CD3 T and CD4 T lymphocytes increased at the second and fourth months compared to baseline. Also, the number of NK cells in the UC-MSC group was higher in the fourth month than at baseline. These results are in line with studies that observed that lymphocyte count returned to the normal range and the time was significantly faster after stromal cell infusion, when compared with the control treatment [49, 121, 122].

The persistent follow-up of discharged patients with COVID-19 is essential to find ways to improve quality of life and reduce morbidity and mortality by efficient prevention. In this study, some markers of cardiac and kidney function were evaluated, and a CT scan was performed for pulmonary evaluation. No differences were observed in relation to troponin I levels, corroborating the results observed by Johnsen et al. [23], who analyzed patients with long COVID-19 sequelae three months post hospitalization and observed no signs of cardiac dysfunction. Creatinine values in the UC-MSC group decreased at the fourth month, while in the placebo group, there was an increase above the reference at the second and fourth months. Kidney lesions acquired during the disease’s activity might remain sequelae that result in a slow and asymptomatic progression toward advanced stages and chronic kidney failure (CKD). Thus, patients who have recovered from COVID-19 who present proteinuria, hematuria, elevated creatinine and AKI should be monitored for CKD [123]. Increased creatinine values may also be associated with the patient's nutritional status [124]; however, these patients underwent nutritional assessment at follow-up, and all of them were in good nutritional status (data not shown).

The benefits of corticosteroid treatment for accelerating the recovery of lung injury, according to pulmonary function assessment and chest imaging in patients with COVID-19, are controversial [73, 125]. Therefore, new strategies to avoid pulmonary sequelae need to be developed. Once injected intravenously, a significant amount of MSCs accumulate in the lungs, and they secrete numerous factors that play an important role in immunomodulation, protect alveolar epithelial cells, restore the pulmonary alveolar niche, prevent fibrosis and improve overall pulmonary function, which is a great benefit for treating severe pulmonary disease in COVID-19 [49, 126]. In addition, lung function and chest CT changes may be impaired months after the infection [127]. Huang et al. [128] observed that a considerable proportion (22–56%) of patients had a pulmonary diffusion abnormality six months after symptom onset. In this trial, there was a decrease in lung lesion extension in the UC-MSC group after four months of follow-up. A similar result was also observed by Shi et al. [58] where the administration of MSC improved in whole-lung lesion volume compared to placebo group and reduced the proportion of solid component lesion volume at each follow-up point, up to 12 months. The improvement of pulmonary lesions directly affects the recovery of lung function and the remission of clinical symptoms [49]; therefore, the results observed in this study could reflect reduced lung inflammation in the UC-MSC group mediated by immune regulation.

Some limitations were faced during this trial, such as the long time elapsed between evaluations, e.g., many parameters may have improved between the day fourteen and 2 months. Sample randomization was conducted in this study; however, based on some inflammation markers and lymphocyte subpopulations, the UC-MSC group seemed to be more compromised than the placebo group at baseline. The sample size was not large enough to stratify subgroups and, thus, bias were difficult to avoid. The emergency condition in ICUs did not allow us to carry out CT evaluations in all patients at different times.

The results of this study revealed that in the UC-MSC group, there was a reduction in the levels of ferritin, IL-6 and MCP1-CCL2 on the fourteen day. In the second month, a decrease in the levels of reactive C-protein was observed, as well as D-dimer and neutrophils and an increase in the numbers of CD3 T, CD4 T and NK lymphocytes were observed. A decrease in extension of lung damage was observed in the fourth month. The improvement in all the parameters was maintained until the end of patient follow-up. Those data show that UC-MSCs treatment for critically ill COVID-19 patients is safe and can play an important role both in the early stages, by preventing *more* severe complications and in the chronic phase, with a reduction in sequelae.

## Conclusions

COVID-19 is a complex multifactorial disease that makes treatment difficult using a single strategy. The promising long-term safety and efficacy results shown in this trial indicate that UC-MSCs could be used as adjunctive therapy for critically ill COVID-19 patients. UC-MSCs showed beneficial effects for patient recovery in the short term through a decrease in CRS by secreting anti-inflammatory factors, reducing risk of thrombosis and, in the long term, via reduction in kidney and pulmonary sequelae based on tissue repair. The combination of immunomodulatory therapy based on UC-MSCs and antiviral drugs could help accelerate patient recovery, attenuating disease progression.

## Supplementary Information

**Additional file 1: Table S1**. UC-MSC Flow cytometry analysis.

**Additional file 2: Table S2**. Group comparison analysis.

**Additional file 3: Table S3**. Longitudinal follow-up.

**Additional file 4: Table S4**. Chest CT features.

## Data Availability

The majority of the data generated or analyzed during this study are included in this article.

## Abbreviations

ARDS: Acute Respiratory Distress Syndrome
AKI: Acute Kidney Injury
ALI: Acute Lung Injury
ATP: Advanced Therapy Product
AE: Adverse Event
ALT: Alanine Aminotransferase
ACE2: Angiotensin-Converting Enzyme 2
ACD: Anticoagulant Citrate Dextrose
PaO_2_: Arterial Oxygen Partial Pressure
AST: Aspartate Aminotransferase
BMI: Body Mass Index
CCT: Core for Cell Technology
CKD: Chronic Kidney Failure
GM‐CSF: Colony‐Stimulating Factor
COVID-19: Coronavirus Disease 2019
CRS: Cytokine Release Syndrome
CT: Computed Tomography
F: Female
FBS: Fetal Bovine Serum
HIV: Immunodeficiency Virus
ICU: Intensive Care Unit
ISCT: International Society for Cellular Therapy
IL: Interleukin
IMDM: Iscove's Modified Dulbecco's Medium
IV: Intravenous Infusion
IMV: Invasive Mechanical Ventilation
MIP1a: Macrophage Inflammatory Protein 1-Alpha
M: Male
MV: Mechanical Ventilation
MSC: Mesenchymal Stromal Cell
MCP1: Monocyte Chemoattractant Protein-1
MOD: Multiple Organ Failure
NK: Natural Killer
NET: Neutrophil Extracellular Trap
FiO_2_: Oxygen Absorption Concentration
P: Passage
PBS: Phosphate Buffered Saline
PUCPR: Pontificia Universidade Católica do Paraná
PCR: Polymerase Chain Reaction
POLR2A: Polymerase II Subunit A
PASC: Postacute Sequelae
RT–PCR: Reverse-Transcription Polymerase Chain Reaction
SARS-Cov-2: Severe Acute Respiratory Syndrome Coronavirus 2
S: Spyke Protein
Tregs: CD4 regulatory T lymphocytes
TMPRSS2: Transmembrane Serine Protease 2
TNF: Tumor Necrosis Factor
UC: Umbilical Cord
UC-MSC: Umbilical Cord Mesenchymal Stromal Cell
WHO: World Health Organization
